# Effects of regional anesthesia techniques on local anesthetic plasma levels and complications in carotid surgery: a randomized controlled pilot trial

**DOI:** 10.1186/s12871-019-0890-8

**Published:** 2019-11-26

**Authors:** Thomas Rössel, Christopher Uhlig, Jörg Pietsch, Stefan Ludwig, Thea Koch, Torsten Richter, Peter Markus Spieth, Stephan Kersting

**Affiliations:** 1 Department of Anaesthesiology and Critical Care Medicine, University Hospital Carl Gustav Carus Dresden, Technische Universität Dresden, Fetscherstr. 74, 01307 Dresden, Germany; 2 Institute of Legal Medicine, Technische Universität Dresden, Dresden, Germany; 3 Department of Visceral, Thoracic and Vascular Surgery, University Hospital Carl Gustav Carus, Technische Universität Dresden, Dresden, Germany; 4 Department of General Surgery, University Hospital of Friedrich-Alexander-University, Erlangen, Germany

**Keywords:** Carotid endarterectomy, Cervical plexus block, Plasma concentration, Regional anesthesia, Local anesthetic, Ropivacaine

## Abstract

**Background:**

The ultrasound guided intermediate cervical plexus block with perivascular infiltration of the internal carotid artery (PVB) is a new technique for regional anesthesia in carotid endarterectomy (CEA). We conducted a pilot study investigating the effects of deep cervical block (DCB), intermediate cervical block alone (ICB) and PVB on perioperative complications in patients undergoing elective CEA. We hypothesized, that the ropivacaine plasma concentration is higher in patients receiving DCB compared to PVB and ICB.

**Methods:**

In a randomized controlled pilot study thirty patients scheduled for elective CEA were randomly assigned into three groups: DCB receiving 20 mL ropivacaine 0.5% (*n* = 10), ICB receiving 20 mL ropivacaine 0.5% (*n* = 10) and PVB receiving 20 mL ropivacaine 0.5% and 10 mL ropivacaine 0,3% (*n* = 10). As primary outcome, plasma levels of ropivacaine were measured with high performance liquid chromatography before, 5, 10, 20, 60, and 180 min after the injection of ropivacaine. Secondary outcomes were vascular and neurological complications as well as patients’ and surgeons’ satisfaction. All analyses were performed on an intention-to-treat basis. Statistical significance was accepted at *p* < 0.05.

**Results:**

No conversion to general anesthesia was necessary and we observed no signs of local anesthetic intoxication or accidental vascular puncture. Plasma concentration of ropivacaine was significantly higher in the DCB group compared to PVB and ICB (*p* < 0.001) and in the PVB group compared to ICB (*p* = 0.008). Surgeons’ satisfaction was higher in the PVB group compared to ICB (*p* = 0.003) and patients’ satisfaction was higher in the PVB group compared to ICB (*p* = 0.010) and DCB group (*p* = 0.029). Phrenic nerve paralysis was observed frequently in the DCB group (*p* < 0.05). None of these patients with hemi-diaphragmatic paralysis showed signs of respiratory distress.

**Conclusion:**

The ultrasound guided PVB is a safe and effective technique for CEA which is associated with lower plasma levels of local anesthetic than the standard DCB. Considering the low rate of complications in all types of regional anesthesia for CEA, larger randomized controlled trials are warranted to assess potential side effects among the blocks.

**Trial registration:**

The trial was registered at German Clinical Trials Register (DRKS) on 04/05/2019 (DRKS00016705, retrospectively registered).

## Background

In carotid endarterectomy (CEA), regional anesthesia is associated with beneficial effects regarding sensitivity and specificity of patients neurological monitoring [1, 2]. CEA in awake patients requires the blockade of cervical nerves from C2 to C4. The blockade can be performed on the nerve roots or on the terminal nerve fibers. The most frequently used regional anesthetic techniques for this purpose are superficial, intermediate and deep cervical block. The anesthetic effects of these three techniques are comparable [3, 4]. However, during dissection of the internal carotid artery (ICA) the need for local anesthetic supplementation by the surgeon ranges from 20 to 60% [5].

Over the last decade, the use of ultrasound has improved the safety and efficacy of regional anesthesia [6, 7]. The major advantages of ultrasound-guided regional anesthesia are the visualization of the target structures, the direct observation of the spread of the local anesthetic and the reduction of puncture-related complications compared to nerve stimulation or land mark technique. Furthermore, new ultrasound-guided anesthetic approaches for blockade of various nerves were developed [8–11]. Our group previously demonstrated a good clinical efficacy with a low rate of intraoperative local anesthetic supplementation by surgeons for the combination of ultrasound-guided intermediate cervical block with perivascular infiltration of the ICA, so called perivascular block (PVB) [8]. On the other hand, due to the vicinity of the vessels the PVB may result on higher plasma levels of local anesthetic compared to ultrasound-guided intermediate cervical block alone (ICB). This may cause more perioperative complications such as dizziness and seizures as described for the deep cervical block (DCB) [4]. In addition to potential toxic effects of local anesthetics, respiratory distress by phrenic nerve paralysis is possible [4].

To our knowledge, ultrasound-guided PVB, ICB and DCB have not been assessed in regard to block performance, perioperative complications and plasma levels of local anesthetics.

Therefore, we investigated the effects of PVB, ICB and DCB on ropivacaine plasma levels, anesthesia related nerve paralysis and efficacy of the block in patients undergoing elective CEA. We hypothesized that the ropivacaine plasma concentration is higher in patients receiving DCB for elective CEA compared to PVB and ICB.

## Methods

**Fig. 1:**
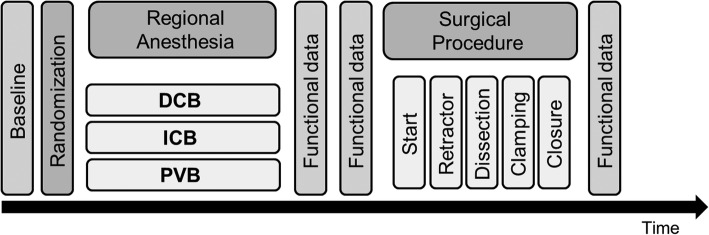
Time course of intervention. DCB: deep cervical block, ICB: intermediate cervical block, PVB: intermediate cervical block with perivascular infiltration of the internal carotid artery

We conducted a randomized controlled, single-center pilot study. The trial is reported according to the Consolidated Standards of Reporting Trials (CONSORT) statement [12]. The experimental protocol is depicted in Fig. 1. After approval by the local institutional review board of the Technische Universität Dresden, Germany (EK 130042013), thirty consecutive patients scheduled for elective CEA in the University Hospital Carl Gustav Carus, Dresden, Germany were screened for eligibility in a 6 month period. Inclusion and exclusion criteria are summarized in Table 1. The patients were randomized directly before start of ultrasound-guided regional anesthesia to three groups: DCB with 20 mL ropivacaine 0.5%, ICB alone with 20 mL ropivacaine 0.5% and combination of intermediate cervical block and perivascular infiltration, PVB, with 20 mL ropivacaine 0.5% and 10 mL ropivacaine 0.3%, respectively. The random sequence was compiled using a computer-generated random numbers table and group allocation was concealed by sequentially numbered opaque closed envelopes. Surgeons and data collectors were blinded to the study group.

**Table 1:** Inclusion and exclusion criteria. CEA: carotid endarterectomy, ICA: internal carotid artery

Inclusion	Exclusion
-age > 18 years -elective CEA surgery for treatment of ICA stenosis -written informed consent	-history of anaphylactic reaction to local anesthetics -local infection in the lateral cervical region -presumed limitation of patients’ compliance

### Regional anesthesia

**Fig. 2:**
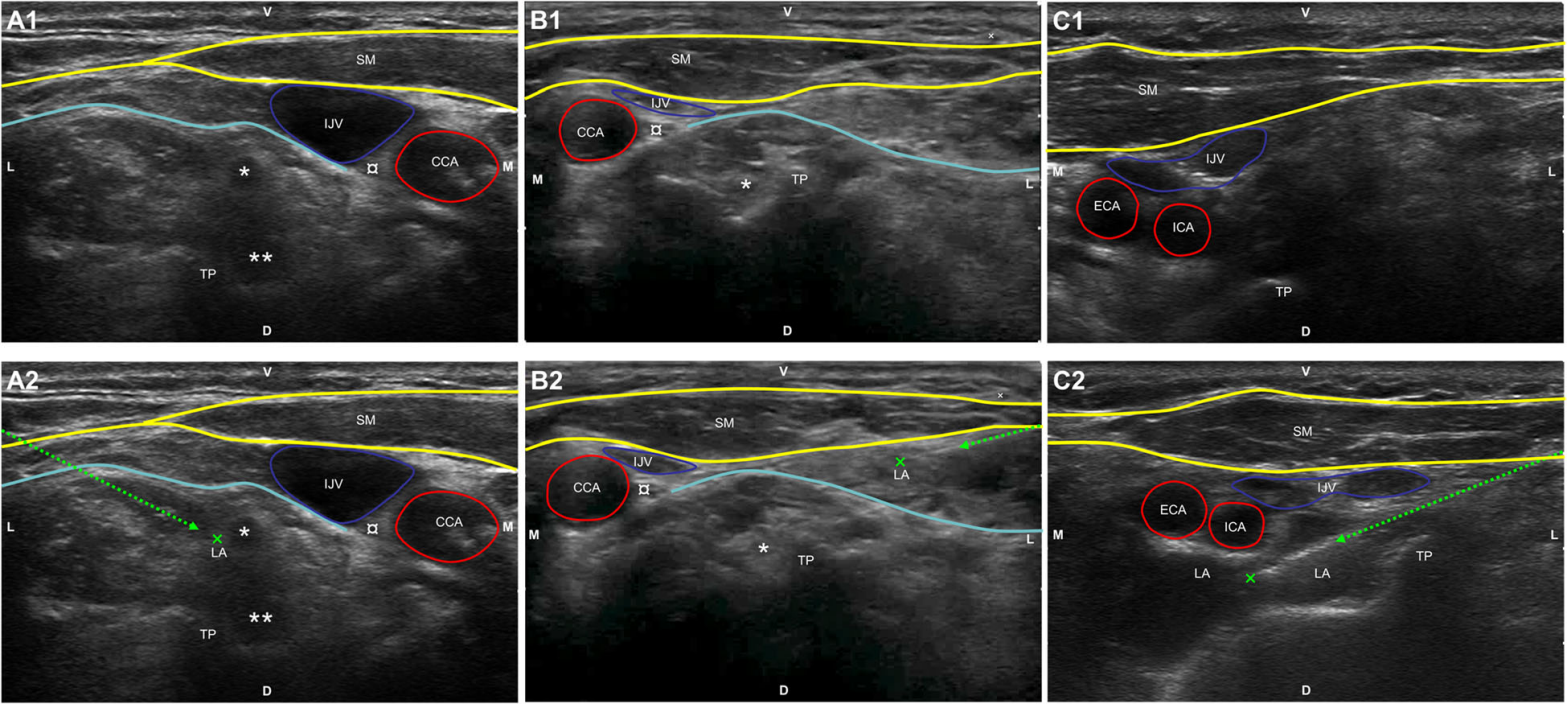
Ultrasound images of cervical block. **a**: deep cervical block, **b**: intermediate cervical block, **c**: intermediate cervical block with perivascular infiltration of the internal carotid artery. Ultrasound images were acquired with Philips HD-11-XE (Philips Healthcare GmbH, Hamburg, Germany using a linear probe (12 MHz, L-12-4, Philips Healthcare GmbH, Hamburg, Germany). Direction of the ultrasound enhanced puncture needle is depicted as green dashed line and the needle tip as green cross. Yellow line: superficial cervical fascia, blue line: deep cervical fascia. LA: local anesthetic, ECA: external carotid artery, ICA: internal carotid artery, IJV: internal jugular vein, CCA: commune carotid artery, TP: Processus transversus of the respective cervical vertebra, V: ventral, D: dorsal, SM: M. steroncleidomastoideus. *: C5 nerve root, **: C6 nerve root, ¤: N. vagus, x: N. auricularis magnus and *N. transversus* colli

Regional anesthesia was performed by two senior anesthesiologists with substantial experience in performance of ultrasound guided deep and intermediate cervical plexus block. The regional anesthetic techniques used in this study were performed as previously described [6, 8]. Briefly, patients were placed for regional anesthesia in supine position with their heads turned 30° to the opposite side. Prior to performing the block, the anatomic conditions of the neck region were analyzed by ultrasound. During this examination, first the transverse process with the corresponding nerve roots from the second to the seventh cervical vertebrae (C2 to C7) as well as the distal part of cervical plexus were visualized and recorded using a Philips HD 11 with a 12.5 MHz linear ultrasound transducer (Philips Medicine Systems GmbH, Hamburg, Germany). Subsequently, the ICA was identified and the distance between the skin and the ICA was recorded. The cervical block was performed according to group allocation (Fig. 2). The success of the blockade was evaluated 5, 10, and 15 min after regional anesthesia by pin prick test in the dermatomes from C2 to C5. Additionally, 20 min after the block was performed, sensory skin testing at the hand, shoulder and motor testing at the wrist, arm and shoulder were performed. Puncture related complications, such as respiratory distress, hypoglossal and facial nerve palsy or Horner’s syndrome were also assessed.

### Intraoperative management and hemodynamic monitoring

The evening before surgery, patients received 25 mg of clorazepate (Aventis GmbH, Bernburg, Germany) per os by request. No premedication was administered at the day of surgery. In the operating room, peripheral venous access, a 5-lead ECG including ST-segment analysis, a pulsoxymetry and an arterial line for continuous monitoring of arterial blood pressure were placed. Hemodynamic data were continuously recorded using a Philips Intellivue MP 70 (Philips Medicine Systems GmbH, Hamburg, Germany). An arterial blood gas analysis was performed before, as well as 15 and 30 min after regional anesthesia. To improve intraoperative comfort, all patients received 0.03 μg/kg/min remifentanil (Aspen-Germany GmbH, Germany; dosage in relation to ideal body weight). After surgery, the patients were observed for 24 h under cardiovascular and neurological monitoring in the intermediate care unit, post anesthesia care unit or regular ward, as appropriate.

### Surgical management

All CEAs were performed by two senior vascular surgeons. Surgery was started when the surgical site had been sufficiently anesthetized. Pain was intraoperatively evaluated by means of the Numeric Analgesia Scale (NAS) graded from 0 (no pain) to 10 (worst pain) during the performance of regional anesthesia and during skin incision, retractor placement, dissection, cross-clamping, and skin closure. If patients complained of intraoperative pain NAS > 2 an additional local infiltration of lidocaine 1% (mibe GmbH, Brehna, Germany) was administered by the surgeon in 1 ml steps until a sufficient anesthetic level was achieved. The total amount of supplemented lidocaine was recorded. At the end of surgery, the surgeons assessed the surgical conditions on a subjective scale ranging from 1 to 5 (1-very good, 2-good, 3-reasonable, 4-poor, 5-very poor). All patients underwent follow-up visits on the first post-operative day. Patients were asked to assess their satisfaction with anesthesia in five grades from 1-very good, 2-good, 3-reasonable, 4-poor to 5-very poor and if they would again undergo surgery under regional anesthesia.

### Plasma level measurement

Arterial blood samples for plasma level of ropivacaine were collected before and 5, 10, 20, 60, and 180 min after the injection of ropivacaine. After immediate centrifugation, plasma samples were stored at − 20 °C. The unbound ropivacaine plasma level was measured by the Institute of Legal Medicine, Dresden Technical University. After a liquid-liquid extraction procedure for sample preparation specimens were analyzed with a high performance liquid chromatography photodiode array detection system (Agilent 1100 series, Agilent Technologies, Waldbronn, Germany). For quantification, drug-free serum was spiked at five different concentrations of ropivacaine (100, 200, 500, 1000, 2000 ng/ml). Ropivacaine concentration was calculated using linear regression. The limit of quantification of the method was 100 ng/ml.

### Assessment of phrenic nerve paresis

The quantitative analysis of phrenic nerve paresis was performed by electrical impedance tomography (EIT, PulmoVista 500, Dräger Medical, Lübeck, Germany) [13]. Images were obtained at baseline, 15, 30 and 180 min after successful establishment of the cervical block. The images containing 32 × 32 pixels were recorded at a rate of 50 frames/s during 2 min for offline analysis. Using a MATLAB (Vers. R2006b, The Mathworks Inc., Natick, MA, USA) based routine, changes in impedance (region of interest – ROI) were determined. The ROI was divided into two zones with equal size corresponding with the left and right lung. Relative changes in impedance were computed. A phrenic nerve paresis was defined as a decrease in change of impedance of more than 50% compared to baseline in one ROI. Phrenic nerve paresis was assessed qualitative and quantitatively by an investigator blinded to the group allocation.

### Neurological monitoring

Neurologic function was perioperatively continuously monitored by observing the level of consciousness and the response to verbal commands. During 5 min test cross-clamping of the ICA, the patient was challenged to squeeze a squeaking rubber toy with the contralateral hand every 10–15 s and to answer simple questions for close judgment of neurological function. A shunt was placed if any signs of neurological dysfunction occurred during test cross-clamping. Additionally, the function of recurrent laryngeal nerve, hypoglossal nerve and facial nerve was monitored before and 30 min after regional anesthetic blockade, as well as before and after cross clamping and at the end of surgery.

### Statistical analysis

Statistical analysis was performed with SPSS (Vers. 20, IBM Deutschland GmbH, Ehningen, Germany). Graphs were computed using Graph Pad Prism Vers. 6.01 (GraphPad Software Inc., La Jolla, CA, USA). Values are given as total numbers and percentage, mean and standard deviation or median and interquartile range as appropriate. Statistical significance was considered at two-sided *p* < 0.05. All analyses were performed on an intention-to-treat basis. Normal distribution was assessed visually using Q-Q Plot of standardized residuals. For the primary outcome, the ropivacaine plasma level, among- and within-groups differences for repeated measures were tested with a general linear model followed by adjustment according to the Sidak method. For secondary outcomes, frequency distributions were analyzed with a Chi-square test followed by a multiple regression approach using adjusted residuals and Bonferoni post hoc test, if appropriate. One-way ANOVA followed by Bonferroni adjustment or Kruskal Wallis test followed by Dunn-Bonferroni test for multiple comparison were used for independent parameters depending on the data distribution. Among- and within-groups differences for repeated measures were tested with a general linear model followed by adjustment according to the Sidak method. Since this study was planned as an explorative trial, no sample size estimation was performed. We opted for 30 patients (10 per group).

## Results

**Fig. 3:**
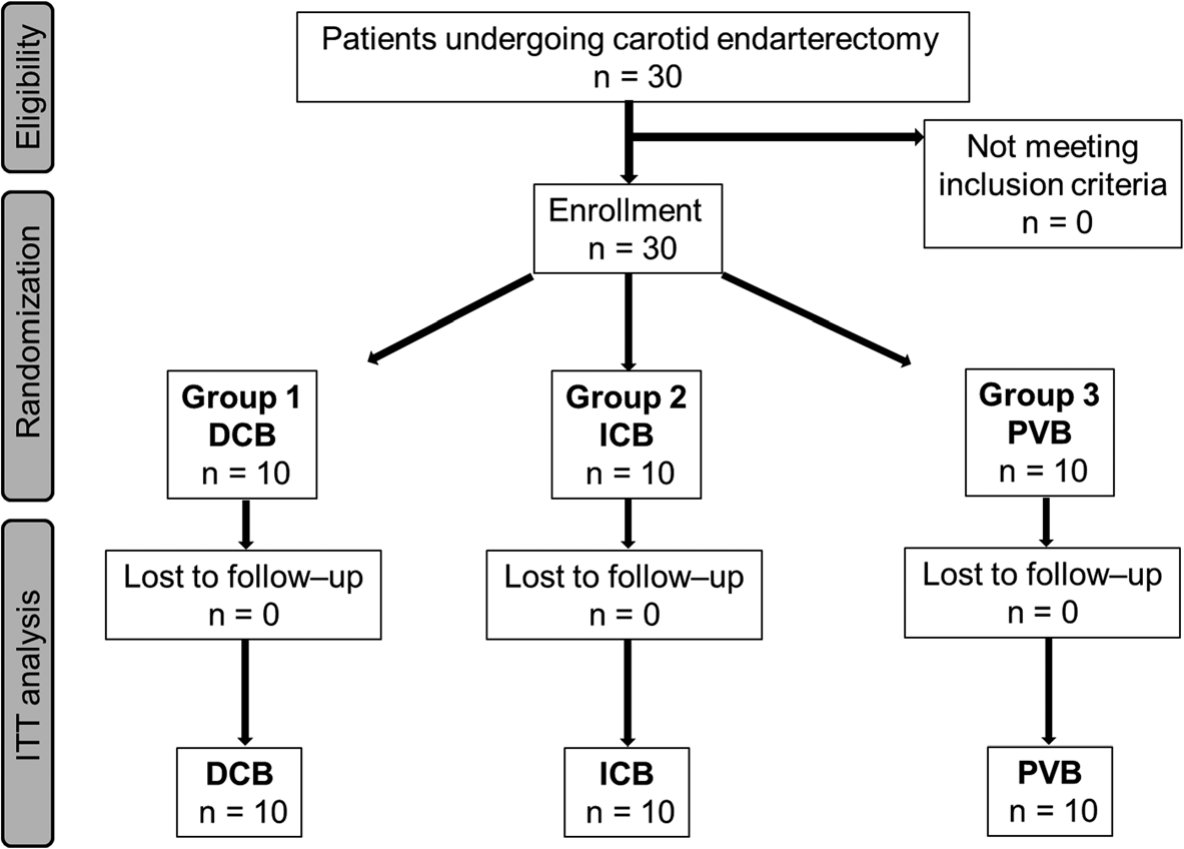
Flow chart of enrolled patients. ITT: intention-to-treat analysis, DCB: deep cervical block, ICB: intermediate cervical block, PVB: intermediate cervical block with perivascular infiltration of the internal carotid artery

Over a period of 7 months, 30 consecutive patients undergoing CEA were enrolled in the trial (Fig. 3). All patients completed the follow-up according to the trial protocol. The three groups were comparable regarding baseline characteristics (Table 2).

**Table 2:** Baseline characteristics

	DCB	ICB	PVB
	(*n* = 10)	(*n* = 10)	(*n* = 10)
Age [years]	67 ± 10	70 ± 9	72 ± 8
Sex male	7 (70.0)	9 (90.0)	8 (80.0)
female	3 (30.0)	1 (10.0)	2 (20.0)
Bodyweight [kg]	72 ± 8	83 ± 8	84 ± 9
Body height [cm]	167 ± 8	174 ± 8	170 ± 6
BMI [kg/m^2^]	25.9 ± 1.6	27.3 ± 2.2	29.1 ± 3.4
Stenosis			
Grade [%]	81 ± 7	82 ± 6	82 ± 9
Sight left	5 (50.0)	5 (50.0)	5 (50.0)
right	5 (50.0)	5 (50.0)	5 (50.0)
asymptomatic	6 (60.0)	6 (60.0)	7 (70.0)
symptomatic	4 (40.0)	4 (40.0)	3 (30.0)
ASA II	6 (60.0)	6 (60.0)	5 (50.0)
III	4 (40.0)	4 (40.0)	5 (50.0)
Comorbidities			
Arterial hypertension	10 (100.0)	10 (100.0)	10 (100.0)
Diabetes mellitus	4 (40.0)	4 (40.0)	5 (50.0)
Hyperlipoproteinema	10 (100.0)	9 (90.0)	10 (100.0)
PAOD	6 (60.0)	1 (10.0)	7 (70.0)
CAD	4 (40.0)	4 (40.0)	5 (50.0)
AMI	1 (10.0)	1 (10.0)	2 (20.0)
Stent	2 (20.0)	2 (20.0)	2 (20.0)
CABG	1 (10.0)	0 (0.0)	3 (30.0)
Artrial fibrillation	1 (10.0)	0 (0.0)	0 (0.0)
COPD	2 (20.0)	2 (20.0)	1 (10.0)
CKD	3 (30.0)	2 (20.0)	1 (10.0)
Nicotine abuse	7 (70.0	7 (70.0	3 (30.0)
Alcohol abuse	5 (50.0)	3 (30.0)	0 (0.0)

Values are given as mean ± standard deviation or absolute number and percentage. *AMI* acute myocardial infarction in medical history, *ASA* American Society of Anesthesiology physic status, *BMI* body mass index, *CAD* coronary artery disease, *CABG* coronary artery bypass graft, *CKD* chronic kidney disease, *COPD* chronic obstructive pulmonary disease, *DCB* deep cervical block, *ICB* intermediate cervical block, *PAOD* peripheral artery occlusive disease, *PVB* intermediate cervical block with perivascular infiltration of the internal carotid artery

### Block execution and performance

**Fig. 4:**
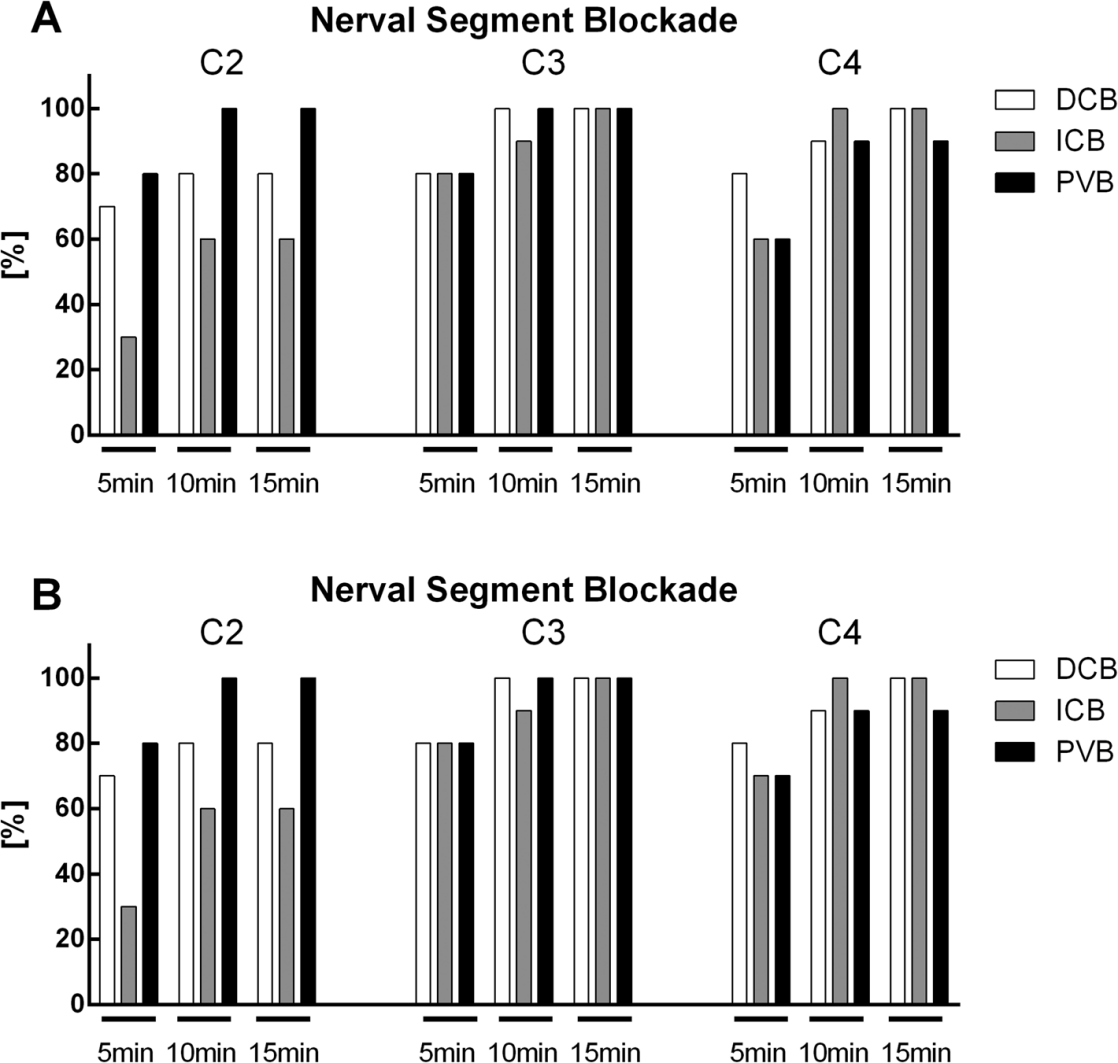
Block distention in the dermatomes C2 to C4. Block distention was determined using peaked/blunt discrimination (**a**) or warm/cold discrimination (**b**). Values are given as percentage and were measured 5 min, 10 min and 15 min after completion of block placement, respectively. DCB: deep cervical block, ICB: intermediate cervical block, PVB: intermediate cervical block with perivascular infiltration of the internal carotid artery

The identification of the nerve roots, of the intermediate cervical plexus, of the ICA and the bifurcation of carotid artery using ultrasound was successful in all patients. The distances from the skin to the ICA was 1.8 ± 0.3 cm in the DCB group, 2.1 ± 0.3 cm in the ICB group and 2.1 ± 0.5 cm in the PVB group (*p* = 0.143). The time required to perform the block was significantly higher in the DCB group compared to PVB (*p* = 0.003, Table 3). The time until full expression of regional anesthesia is depicted in Fig. 4. All three groups showed sufficient analgesia in the dermatomes C3 and C4, but PVB was the only block providing analgesia in the dermatome C2 in all patients.

**Table 3:** Supplementation of block with additional local anesthesia by the surgeon and NAS results (0 = no pain −10 = worst imaginable pain)

		DCB	ICB	PVB	*P* value
		(*n* = 10)	(*n* = 10)	(*n* = 10)	
Duration of block disposition [min]		15.9 ± 2.8	11.5 ± 2.1	14.1 ± 2.0	0.019*
Additional lidocaine [No.,%]		6 (60.0)	8 (80.0)	4 (40.0)	n.a.
Additional lidocaine [mg]		90.0 (50.0, 100.0, 145.0,160.0)	150.0 (50.0, 100.0, 200.0, 300.0)	85.0 (30.0, 35.0, 120.0,120.0)	n.a.
Numeric analog scale*	RA	2 (0, 2, 4, 5)	3 (0, 1, 3, 5)	2 (1, 1, 2, 3)	0.336
	Incision	1 (0, 1, 3, 5)	2 (0, 1, 2, 5)	1 (0, 1, 2, 3)	0.659
	Retractor	1 (0, 1, 2, 4)	2 (0, 1, 3, 3)	1 (0, 1, 2, 3)	0.301
	Dissection	2 (1, 1, 4, 6)	1 (0, 3, 4, 5)	2 (1, 2, 2, 3)	0.061
	Clamping	2 (0, 1, 2, 2)	2 (0, 2, 3, 3)	2 (1, 2, 3, 5)	0.153
	Suture	1 (0, 1, 1, 5)	2 (0, 1, 2, 3)	1 (0, 1, 2, 2)	0.189

Values are given as mean ± standard deviation, median (minimum, 25% percentile, 75% percentile, maximum) or absolute number (percentage) as appropriate. Differences among groups were tested with Kruskal-Wallis followed by Dunn-Bonferroni test. Statistical significance was considered to be at two-sided *p* < 0.05. *DCB* deep cervical block, *ICB* intermediate cervical block, *PVB* intermediate cervical block with perivascular infiltration of the internal carotid artery, *RA* regional anesthesia, *No* number, *n.a* statistics not applicable due to low patient number in the PVB group. *: *p* < 0.001 DCB vs. ICB

**Fig. 5:**
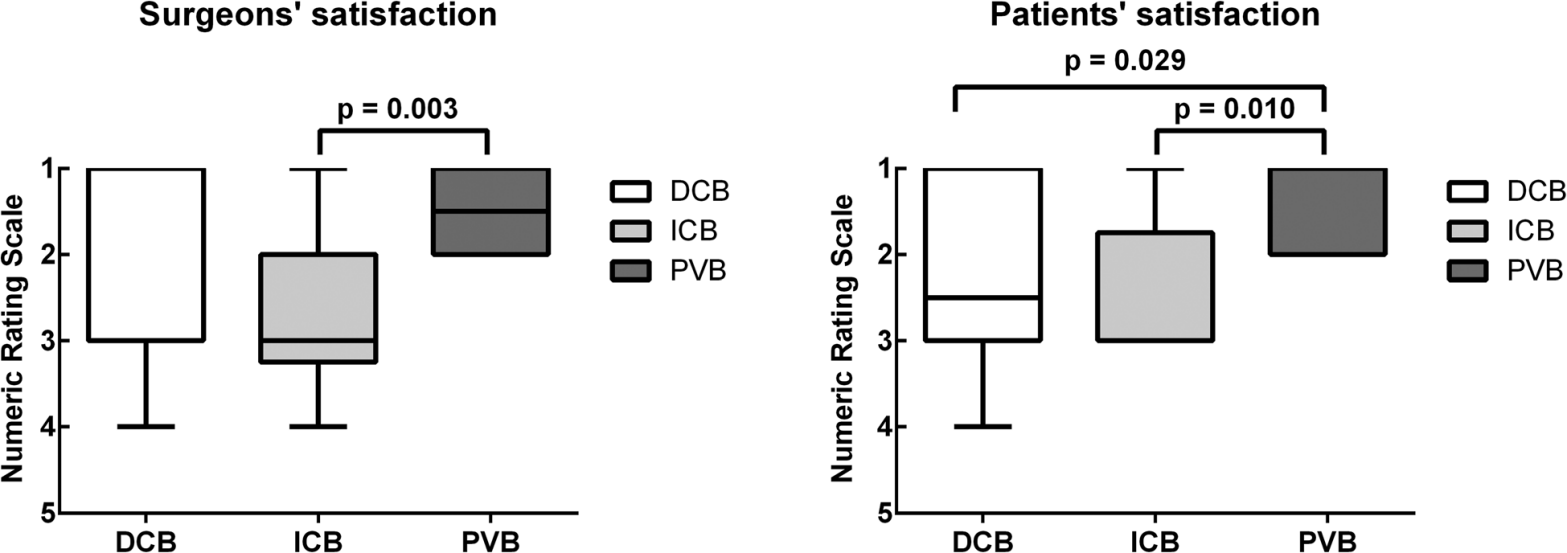
Surgeons’ and Patients’ satisfaction. Values are presented as boxplot boxplot (whiskers minimum to maximum) on a numeric rating scale. Grade system: 1-very good, 2-good, 3-reasonable, 4-poor, 5-very poor. Statistical analysis was performed using Kruskal Wallis test followed by Dunn-Bonferroni test for multiple comparison. Statistical significance was considered to be at two-sided *p* < 0.05. DCB: deep cervical block, ICB: intermediate cervical block, PVB: intermediate cervical block with perivascular infiltration of the internal carotid artery

During surgery, 18 patients complained about pain NAS ≥ 2 resulting in supplementation of the block with local lidocaine 1% by the surgeon (6 vs. 8 vs. 4 patients, DCB vs. ICB vs. PVB, *p* = 0.189, respectively). In the PVB group a lower dosage of supplementation was required (Table 3). No conversion to general anesthesia due to an incomplete block or any other reasons was necessary. The duration of surgery was 103 ± 33 mins in the DCB group, 103 ± 17 mins in the ICB and 107 ± 21 min in the PVB group (*p* = 0.874). The cross-clamping time was 37 ± 10 min in the DCB group, 37 ± 9 min in the ICB group and 29 ± 5 min in group with PVB (*p* = 0.061). Planned or unplanned shunt placement was not necessary in any case. Surgeons’ satisfaction was higher in the PVB group compared to ICB and patients’ satisfaction was higher in the PVB group compared to ICB and TCB group (Fig. 5). Hemodynamic and functional data as well as cardiac biomarkers are shown in Additional file 1: Tables S1, S2 and Fig. S1. The length of hospital stay was 7.7 ± 4.7 days in the DCB group, 5.2 ± 1.1 days in the ICB group and 5.3 ± 0.9 days in the PVB group (*p* = 0.610). There were no in-hospital deaths observed.

### Ropivacaine plasma concentration

**Fig. 6:**
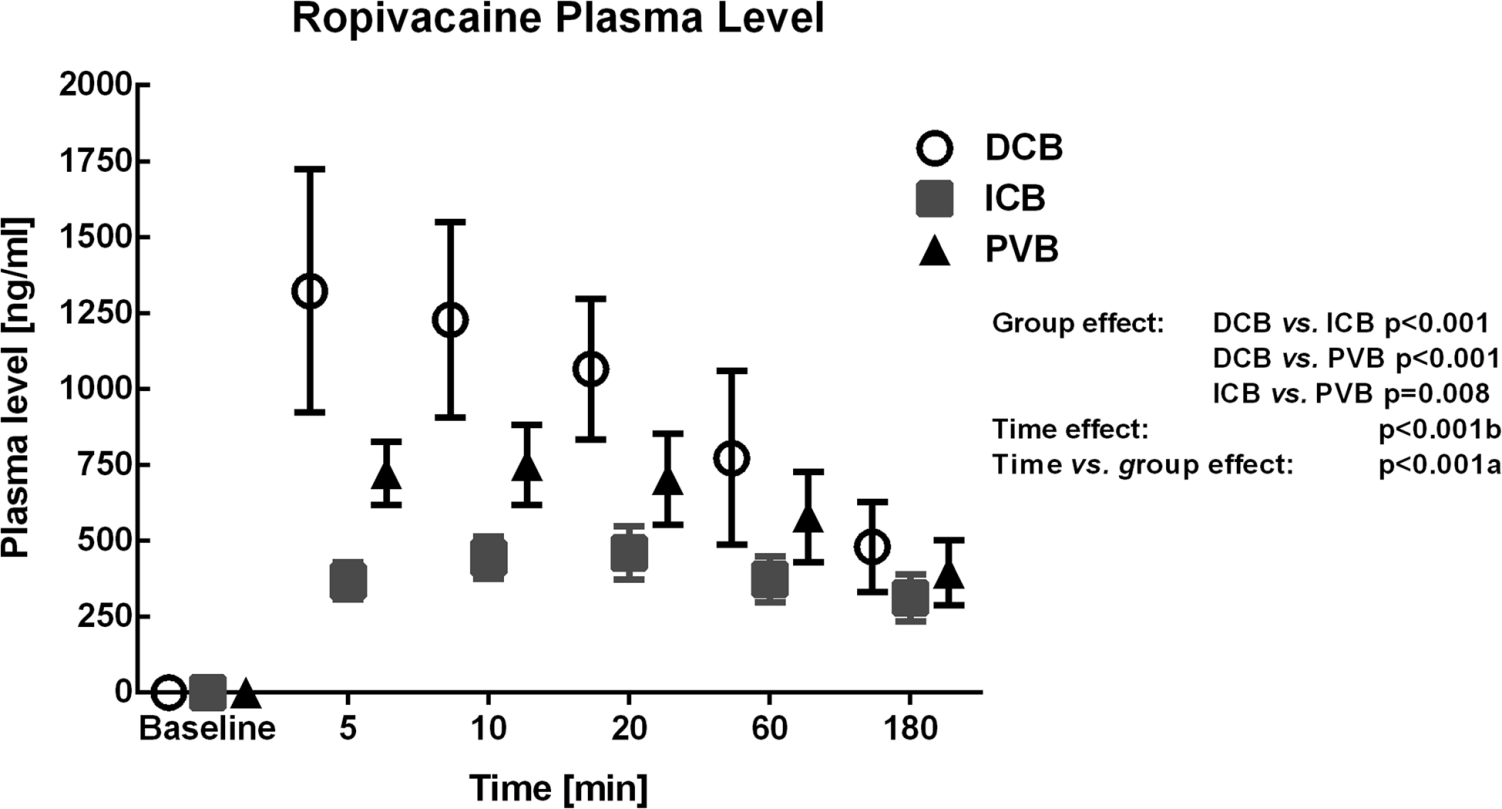
Ropivacaine plasma concentration. Values are given as mean ± standard deviation. Differences among groups, as well as time and time vs. group effects were tested using a general linear model without a covariate. Statistical significance was considered to be at two-sided *p* < 0.05. DCB: deep cervical block, ICB: intermediate cervical block, PVB: intermediate cervical block with perivascular infiltration of the internal carotid artery

The ropivacaine plasma concentration is shown in Fig. 6. The plasma concentration of ropivacaine was significantly higher in the DCB and PVB group compared to the ICB group (DCB vs. ICB, *p* < 0.001; DCB vs. PVB, *p* = 0.001; ICB vs. PVB, *p* = 0.008; respectively). There was no adverse event related to the systemic plasma level of ropivacaine in all groups*.*

### Neurological complications

**Fig. 7:**
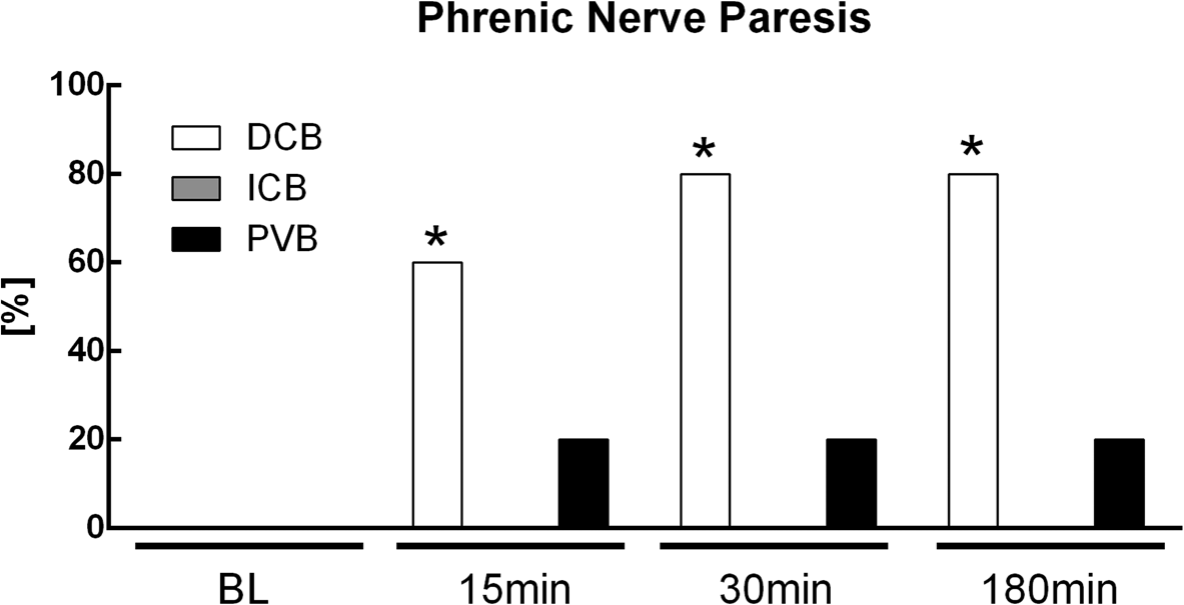
Incidence of phrenic nerve paralysis. Values are given as percentage at baseline (BL), 5 min, 30 min, and 180 min after completion of block placement, respectively. A Chi-square test with multiple regression approach and Bonferroni post hoc test were performed. Statistical significance was accepted a *p* < 0.05. DCB: deep cervical block, ICB: intermediate cervical block, PVB: intermediate cervical block with perivascular infiltration of the internal carotid artery. *: *p* < 0.05

No patient suffered from new intra- or postoperative central neurological deficits. Horner syndrome, hypoglossal nerve or permanent facial nerve paralysis were not observed in any patient. Hemi-diaphragmatic impairment caused by phrenic nerve paralysis was associated with a more frequent occurrence in the DCB group (*p* = 0.022, Fig. 7). None of these patients with hemi-diaphragmatic paralysis showed signs of respiratory distress.

## Discussion

### Major findings

The major findings of the present study are:

1. The plasma level of local anesthetic was significantly higher in the DCB group and PVB group compared to ICB alone, without causing adverse events.
2. Impairment of ventilation due to hemi-diaphragmatic paralysis was frequently observed in the DCB group.
3. The PVB is a feasible regional anesthetic technique providing sufficient analgesia for CEA in all of the desired dermatomes C2-C4.

This is the first trial comparing ropivacaine plasma levels with PVB, DCB and ICB. Regional anesthesia and CEA were performed by senior physicians. We tried to reduce bias by blinding the patients, the surgeons and outcome assessors to groups. In addition to improve adherence to blinding, the anesthesiologist performing the cervical block did not treat the patient during surgery. Adherence to allocation concealment and blinding of participants, study personnel and outcome assessors were maintained throughout the trial.

### Regional anesthesia in CEA

The implementation of ultrasound in regional anesthesia has increased safety and efficacy by direct visualization of the target structure and the needle tip as well as by observation of local anesthetic spread during injection. Despite these advantages, even the ultrasound guided regional anesthetic techniques require a high level of local anesthetic infiltration by surgeons [14–16]. In our opinion, an important reason for the high rate of local anesthetic supplementation is the complex innervations of the neurovascular sheath by the vagal and glossopharyngeal nerve. In several previous studies, ultrasound guided perivascular infiltration of the ICA decreased the necessity of local anesthetic supplementation by surgeons and increased the efficacy of regional anesthesia [8, 17, 18]. However, the influence of perivascular infiltration on local anesthetic plasma concentration or the risk of phrenic nerve paralysis were not compared so far.

### Ropivacaine plasma levels

The plasma concentration of local anesthetic depends on different conditions. Particularly, the type of local anesthetic as well as the vascularization of the puncture site are important factors of local anesthetic absorption. Ropivacaine is associated with low lipid solubility and provides a better neurological and cardiac toxicity profile than bupivacaine. Additionally, the vasoconstrictive effetcs of ropivacaine delays the absorption of the local anesthetic and may therefore be particularly suitable for regional anesthetic techniques in highly vascularized regions. Besides this advantages also for ropivacaine severe complications up to local anesthetic intoxications with cardiac arrest are reported [19, 20].

In carotid endarterectomy cerebral seizure by local anesthetic intoxication can lead to the inability to properly monitor neurological symptoms and increases the oxygen consumption of the brain. Davies et al. reported two cases of local anesthetic intoxications in 1000 carotid endarterectomies, which equals an incidence of 0.2% [5]. The occurrence of cerebral symptoms depends on the maximum ropivacaine plasma concentration as well as the slope of the plasma level increase.

In the present study, the lowest peak concentrations of ropivacaine (0.3 μg/mL) were measured in the intermediate cervical block group and the highest peak concentrations (2.1 μg/mL) in the deep cervical block group. However, the detected ropivacaine plasma concentrations were well below the threshold for early neurological toxicity symptoms to be 2.2 μg/mL as described by Knudsen et al. [21]. Different groups reported comparable plasma concentrations of ropivacaine after interscalene or deep cervical plexus block [22]. In contrast, few studies examined the ropivacaine plasma concentrations after intermediate cervical block. Koköfer et al. reported plasma concentrations after ultrasound guided triple injection technique (intermediate cervical block, perivascular infiltration and subcutane infiltration) [7]. This group used 20 mL ropivacaine 0.375% or 0.75% for intermediate cervical block and prilocaine 1% for the perivascular infiltration of ICA. Additionally, prilocaine 1% was used also for the subcutaneous infiltration along the anterior border of sternocleidomastoid muscle. The peak plasma levels in the ropivacaine 0.375% group ranged from 4 to 7 μg/mL and in the ropivacaine 0.75% group from 5 to 10 μg/mL. In contrast, the peak plasma levels for intermediate cervical block in our study ranged from 0.3 to 0.6 μg/mL. Several reasons may explain these different results of local anesthetic plasma levels after intermediate cervical plexus block. The use of two different local anesthetics by Koköfer could have led to an increase of ropivacaine plasma concentration. Another cause might be the binding of ropivacaine to α_1_-acid glycoprotein, which may markedly affect the pharmacokinetics of ropivacaine [23]. However, α_1_-acid glycoprotein levels were measured neither in the present study, nor by Koköfer [7].

The effects of perivascular infiltration on ropivacaine plasma concentration was not examined in any study. Although Koköfer et al. performed a perivascular infiltration, the effect on plasma concentration was not assessed. He used prilocaine 1% for perivascular infiltration as well as for subcutaneous infiltration. In our study, we applied only ropivacaine for all regional anesthetic techniques. The ropivacaine plasma concentrations of the perivascular group were significantly higher than for intermediate block alone. In our opinion, the reason of the higher ropivacaine concentration is the larger volume of local anesthetic applied in the perivascular group compared to the intermediate block alone suggesting similar tissue adsorption characteristics.

The threshold plasma concentration at which central nerve system toxicity occurs may be related more to the rate of increase of the serum concentration rather than to the total amount of drug injected. Wulf and colleagues examined the plasma concentration after combined ilioinguinal-iliohypogastric block with ropivacaine [24]. The peak plasma concentrations of ropivacaine were 1.5 μg/mL and occurred 45 min after injection. In contrast, Rettig et al. examined the plasma concentrations of ropivacaine after brachial plexus blockade using four different approaches [22]. The authors reported the lateral and posterior interscalene block to be associated with earlier (10 and 15 min, respectively) and high peak plasma concentrations of local anesthetic (4,4 μg/mL and 4,5 μg/mL). According to these studies, the increase in plasma concentrations is significantly influenced by the anatomic region of regional anesthesia. In the present study, the fastest increase in ropivacaine concentration was observed in the deep cervical block group. However, no cerebral signs of local anesthetic intoxication were observed. The time to reach the maximum concentration was 5 to 10 min. Similar results were reported by Merle, who found times of 5 to 17 min for the classic deep cervical block [25]. In contrast to deep cervical block, in our study the increase in ropivacaine plasma concentrations was significantly slower (10 to 20 min) for intermediate cervical plexus block alone. Similar results were observed for combination of intermediate cervical block and perivascular infiltration. In this group the time to reach the maximum concentration was 10 to 20 min. In our opinion, the reason of the faster increase in ropivacaine plasma concentration is a pronounced vascularization in the deep cervical space.

### Phrenic nerve paralysis

Phrenic nerve paralysis can occur during cervical block due to the close anatomical relation. The phrenic nerve origins mainly from the C4 root, with variable portions from the C3 and C5 root [26]. After formation of the phrenic nerve at the upper lateral border of the anterior scalene muscle the nerve continues caudally between the ventral surface of the anterior scalene muscle and prevertebral fascial layer that covers this muscle and is therefore separated from the brachial plexus only by a thin fascial layer [26]. During regional anesthesia, a perioperative phrenic nerve paralysis can have various causes [27, 28]. Temporary phrenic nerve palsies are most common after cardiac surgery but may also be caused by CEA due to traction or compression as well as local anesthetic supplementation [29].

In the current trial, all patients showed bilateral ventilation before regional anesthesia. In ten patients (DCB: *n* = 8, PVB: *n* = 2) a phrenic nerve paresis was observed. None of these patients suffered from respiratory distress. The high rate of phrenic nerve paralysis in our investigation is not surprising for deep cervical block, where a phrenic nerve paralysis occur in 55 to 61% of the cases [30, 31]. An even higher rate up to 100% of hemi-diaphragmatic paralysis is reported by Urmey et al. for the interscalene brachial plexus block [32, 33]. Despite this high incidence of paralysis of the phrenic nerve, reports of significant shortness of breath or impairment of gas exchange are rare [33–35]. The occurrence of phrenic nerve paresis in the perivascular group is more difficult to explain. The precise anatomy of the deeper neck compartments is complex and has not been completely understood so far. For decades, the concept of impenetrability of the deep fascia of the neck for local anesthetics was indefeasible [36], but has been questioned recently [37, 38]. These doubts are supported by case reports observing complications such as Horner syndrome after superficial blocks [39]. Furthermore, Pandit et al. described in a cadaver study penetration of a superficial injection of methylene blue to the nerve roots in the deep space [40]. Contrary, in another corpse study, Seidel and colleagues observed no spread of methylene blue through the deep cervical fascia [36]. Nevertheless, there were clear methodological differences between these two cadaver studies, especially in the fluid volume administered [36, 40]. In our opinion, larger volumes of local anesthetic may cause higher intra-compartment pressures and therefore enhance a deeper spread of the local anesthetic via the anatomic pathways described by Pandit [38, 40]. This may result in phrenic nerve paresis in the deep cervical compartment. However, further detailed studies are warranted to prove this hypothesis.

### Limitations

The present trial has several limitations. First, the present trial was an explorative pilot study. Therefore, no sample size calculation was performed. Second, the hemi-diaphragmatic paresis was diagnosed indirectly through decrease of regional ventilation in one lung via EIT. This functional approach to phrenic nerve paresis was described by Reske and colleagues for interscalene brachial plexus block in a small patient collective [13]. The EIT has the major advantage that the impairment of ventilation could easily be detected at the bedside [41]. Ultrasound imaging of the diaphragm is more observer dependent and can be difficult in obese patients. In contrast, the EIT has also been used in obese patients by Nestler et al. [42]. However, the EIT method for detecting hemi-diaphragmatic paresis has not been validated in a larger patient collective so far. Parallel ultrasound imaging of the diaphragm was not performed in the current trial. Third, the assessment of patient and surgeon satisfaction with the respective block was subjective. The simple grading scale for satisfaction from 1 to 5 was chosen for patients’ feasibility. The same grading was used for surgeons’ rating with regards to comparability. Fourth, the individual patient pain and convenience level in the operating room may have influenced the surgeons’ decision for additional administration of local anesthetic in the operating situs.

### Implications for further studies

Future trials investigating the effects of different regional anesthetic techniques such as DCB, ICB and PVB on patient safety, systemic local anesthetic concentration and side effects are warranted. Such a trial should be prospective, randomized, controlled and ideally triple blind focusing for instance on postoperative pulmonary complications caused by phrenic nerve paralysis with dual assessment of diaphragm function by EIT and ultrasound as primary outcome. The present trial may provide a basis for sample size calculation. However, the evaluation of systemic toxic side effects of local anesthetics during regional anesthesia for CEA will be difficult due to the rare occurrence [5]. A prospective observational trial focusing on the occurrence of seizures and new arrhythmias in context to the regional anesthetic technique may help to further investigate these systemic and clinical relevant side effects. The adaptation of the data entry in national or international databases for CEA regarding the specific regional anesthetic technique and type of anesthetic may help researchers to get access to a larger data set for a retrospective trial.

## Conclusions

The ultrasound guided intermediate cervical block with perivascular infiltration of the internal carotid artery is a safe and feasible technique for carotid endarterectomy. However, further studies in a larger patient collective are warranted to evaluate rare side effects related to the area of administration and systemic plasma concentration of the local anesthetic.

## Supplementary information

**Additional file 1: Figure S1.** Cardiac biomarkers. **Table S1.** Hemodynamic data and **Table S2.** Blood gas analysis data.

## Data Availability

The datasets used and/or analyzed during the current study are available from the corresponding author on request.

## Abbreviations

CEA: Carotid endarterectomy
CONSORT: Consolidated Standards of Reporting Trials
DCB: Deep cervical block
EIT: Electrical impedance tomography
ICA: Internal carotid artery
ICB: Intermediate cervical block
PVB: Intermediate cervical block with perivascular infiltration of the internal carotid artery
ROI: Region of interest
