# Supplementary material for: Effects of regional anesthesia techniques on local anesthetic plasma levels and complications in carotid surgery: a randomized controlled pilot trial
Source: BMC Anesthesiol. 2019 Nov 26;19:218. doi: 10.1186/s12871-019-0890-8 (PMC6878670; doi:10.1186/s12871-019-0890-8)
Supplement: Supplementary file 1 — Additional file 1: Figure S1. Cardiac biomarkers. Table S1. Hemodynamic data and Table S2. Blood gas analysis data. [file 12871_2019_890_MOESM1_ESM.pdf]

Additional File 1

Effects of regional anesthesia techniques on local anesthetic plasma levels and complications in carotid surgery: a randomized controlled pilot trial.

Thomas Rössel[1], Christopher Uhlig[1], Jörg Pietsch[2], Stefan Ludwig[3], Thea Koch[1], Torsten Richter[1], Peter Markus Spieth[1], Stephan Kersting[3,4]

[1]Department of Anaesthesiology and Critical Care Medicine, University Hospital Carl Gustav Carus Dresden, Technische Universität Dresden, Dresden, Germany, [2]Institute of Legal Medicine, Technische Universität Dresden, Dresden, Germany, [3]Department of Visceral, Thoracic and Vascular Surgery, University Hospital Carl Gustav Carus, Technische Universität Dresden, Dresden, Germany, [4]Department of General Surgery, University Hospital of Friedrich-Alexander-University, Erlangen, Germany.

Drs. Rössel and Uhlig contributed equally to this manuscript.

Corresponding author:

Thomas Rössel, MD

Department of Anaesthesiology and Critical Care Medicine

Universitätsklinikum Carl Gustav Carus Dresden

Technische Universität Dresden

Fetscherstr. 74

01307 Dresden, Germany

phone: +49 351 458 2785 fax: +49 351 458 4336
email: thomas.roessel@ukdd.de

Table of content

Figure S1……………………………………………………………………………………………….3

Table S1………………………………………………………………………………………………..4

Table S2………………………………………………………………………………………………..5

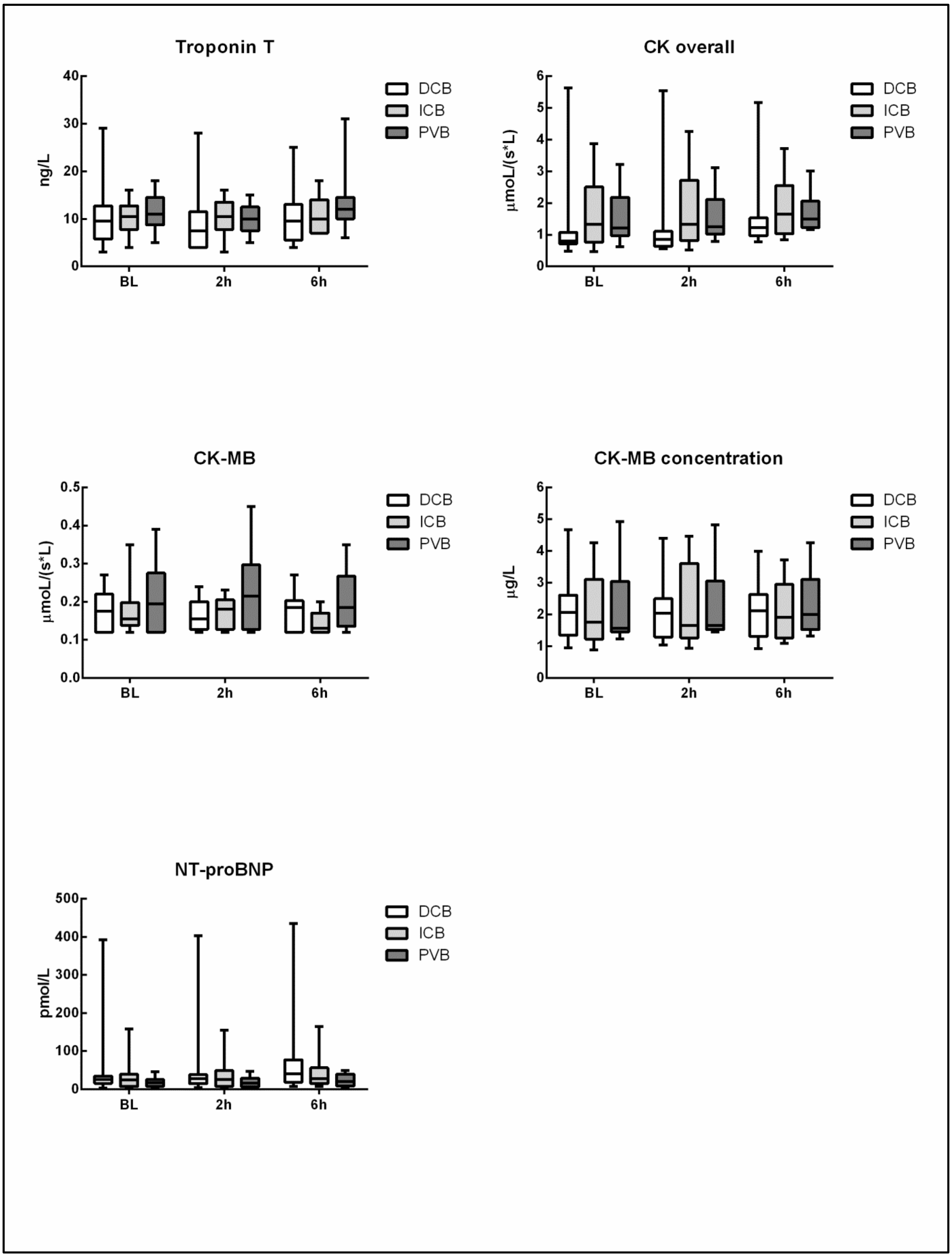

Figure S2: Cardiac biomarkers. Values are given as boxplot (whiskers minimum to maximum). Differences among groups were tested with Kruskal-Wallis followed by Dunn's multiple comparison test. Statistical significance was considered to be at two-sided $p < 0.05$. DCB: deep cervical block, ICB: intermediate cervical block, PVB: intermediate cervical block with perivascular infiltration of the internal carotid artery. CK: creatine kinase, CK-MB: creatine kinase-MB isoenzyme, NT-proBNP: N-terminal prohormone of brain natriuretic peptide.

Table S1

		BL	RA	5 min	10 min	20 min	30 min	SoS	Dissection	Clamping	EoS	P value
**RRmean**	DCB	110 ± 17	115 ± 18	122 ± 20	117 ± 17	115 ± 16	118 ± 21	112 ± 19	112 ± 10	127 ± 17	100 ± 10	Group: 0.428
**[mmHg]**	ICB	99 ± 14	102 ± 13	104 ± 15	105 ± 11	106 ± 11	107 ± 18	103 ± 11	106 ± 7	123 ± 16	99 ± 19	Time: 0.084
	PVB	105 ± 18	98 ± 33	115 ± 11	115 ± 10	111 ± 10	116 ± 14	117 ± 12	113 ± 7	120 ± 9	100 ± 8	Group*Time: 0.442
**RRsys**	DCB	170 ± 28	174 ± 29	185 ± 31	181 ± 29	179 ± 31	177 ± 30	169 ± 26	176 ± 15	190 ± 22	148 ± 12	Group: 0.716
**[mmHg]**	ICB	151 ± 23	157 ± 23	161 ± 24	162 ± 21	166 ± 22	165 ± 27	161 ± 17	164 ± 15	189 ± 14	152 ± 20	Time: <0.001a
	PVB	170 ± 20	168 ± 18	186 ± 16	184 ± 17	181 ± 15	177 ± 21	182 ± 15	180 ± 16	189 ± 9	153 ± 15	Group*Time: 0.511
**RRdias**	DCB	77 ± 10	78 ± 10	83 ± 13	84 ± 12	83 ± 11	82 ± 13	80 ± 14	80 ± 10	87 ± 11	70 ± 9	Group: 0.412
**[mmHg]**	ICB	71 ± 9	71 ± 6	72 ± 7	74 ± 7	74 ± 7	75 ± 7	74 ± 10	76 ± 4	84 ± 9	72 ± 9	Time: 0.106
	PVB	75 ± 13	72 ± 10	81 ± 14	79 ± 12	76 ± 11	76 ± 14	77 ± 13	81 ± 11	80 ± 11	68 ± 7	Group*Time: 0.354
**HF [bpm]**	DCB	74 ± 13	73 ± 12	77 ± 11	76 ± 12	77 ± 13	77 ± 10	75 ± 14	78 ± 12	83 ± 11	81 ± 11	Group: 0.593
	ICB	65 ± 6	65 ± 5	70 ± 6	68 ± 6	71 ± 8	67 ± 9	68 ± 12	69 ± 11	77 ± 14	77 ± 6	Time: 0.005a
	PVB	72 ± 10	72 ± 9	77 ± 13	77 ± 11	75 ± 11	78 ± 12	79 ± 13	77 ± 12	83 ± 9	79 ± 10	Group*Time: 0.639

Table S1 - Hemodynamic data. Values were measured at baseline (BL) as well as during cervical block placement (RA), 5mins, 10min, 20min and 30min after completion of regional anesthesia, start of surgery (SoS), Dissection, Clamping and end of surgery (EOS), respectively. Values are given as mean ± standard deviation. Differences among groups as well as time and time vs. group effect were analyzed using a general linear model with adjustment according to Sidak. Statistical significance was considered to be at two-sided $p < 0.05$. a: linear effect, RRmean: mean arterial blood pressure, RRsys: systolic arterial blood pressure, RRdias: diastolic arterial blood pressure, HF: heart frequency, bpm: beats per minute, DCB: deep cervical block, ICB: intermediate cervical block, PVB: intermediate cervical block with perivascular infiltration of the internal carotid artery.

Table S2

		BL	15min	30min	180min	P value
**PaO_2 [kPa]**	DCB	9.9 ± 0.9	9.6 ± 1.7	10.0 ± 2.2	12.1 ± 5.1	Group: 0.751
	ICB	10.4 ± 1.1	10.1 ± 1.5	10.4 ± 1.8	10.9 ± 2.6	Time: 0.055
	PVB	10.3 ± 1.4	9.5 ± 1.4	10.1 ± 1.7	13.9 ± 8.1	Group*Time: 0.463
**$PaCO_2$ [kPa]**	DCB	4.5 ± 0.6	4.8 ± 0.4	5.0 ± 0.5	4.9 ± 0.8	Group: 0.350
	ICB	4.6 ± 0.3	4.7 ± 0.5	4.5 ± 0.6	5.1 ± 0.8	Time: 0.350
	PVB	4.7 ± 0.6	5.2 ± 0.9	5.1 ± 0.8	5.4 ± 1.0	Group*Time: 0.240
**pH**	DCB	7.45 ± 0.04	7.44 ± 0.03	7.43 ± 0.03	7.41 ± 0.05	Group: 0.736
	ICB	7.46 ± 0.06	7.43 ± 0.04	7.45 ± 0.04	7.38 ± 0.05	Time: 0.096
	PVB	7.43 ± 0.04	7.43 ± 0.11	7.42 ± 0.04	7.37 ± 0.06	Group*Time: 0.377
**Hb [mmol/L]**	DCB	8.6 ± 1.0	8.6 ± 1.0	8.6 ± 1.0	8.3 ± 0.9	Group: 0.973
	ICB	8.5 ± 0.4	8.4 ± 0.4	8.4 ± 0.4	8.3 ± 0.4	Time: 0.405
	PVB	8.8 ± 0.6	8.8 ± 0.6	8.7 ± 0.8	8.4 ± 0.8	Group*Time: 0.196
**Hct [%]**	DCB	0.43 ± 0.05	0.43 ± 0.05	0.42 ± 0.05	0.41 ± 0.05	Group: 0.285
	ICB	0.42 ± 0.02	0.41 ± 0.02	0.41 ± 0.02	0.41 ± 0.02	Time: 0.347
	PVB	0.43 ± 0.04	0.43 ± 0.03	0.43 ± 0.04	0.42 ± 0.04	Group*Time: 0.261
**BS [mmol/L]**	DCB	6.6 ± 1.0	6.7 ± 1.1	6.7 ± 1.3	8.3 ± 1.5	Group: 0.558
	ICB	7.4 ± 2.0	7.4 ± 1.9	7.3 ± 1.9	9.2 ± 2.5	Time: 0.009a
	PVB	8.5 ± 3.0	8.2 ± 2.4	8.6 ± 2.4	10.5 ± 2.2	Group*Time: 0.578

Table S2 - Blood gas analysis data. Values were measured at baseline (BL) as well as 15mins, 30min, and 180min after completion of regional anesthesia, respectively. Values are given as mean ± standard deviation. Differences among groups as well as time and time vs. group effect were analyzed using a general linear model with adjustment according to Sidak. Statistical significance was considered to be at two-sided $p < 0.05$. a: linear effect, PaO_2: arterial oxygen partial pressure, $PaCO_2$: arterial carbon dioxide partial pressure, pH: pondus hydrogenii, Hb: hemoglobin, Hct: hematocrit, BS: blood sugar, DCB: deep cervical block, ICB: intermediate cervical block, PVB: intermediate cervical block with perivascular infiltration of the internal carotid artery.
